# Interleukin-1β in tendon injury enhances reparative gene and protein expression in mesenchymal stem cells

**DOI:** 10.3389/fvets.2022.963759

**Published:** 2022-08-11

**Authors:** Drew W. Koch, Alix K. Berglund, Kristen M. Messenger, Jessica M. Gilbertie, Ilene M. Ellis, Lauren V. Schnabel

**Affiliations:** ^1^Department of Clinical Sciences, College of Veterinary Medicine, North Carolina State University, Raleigh, NC, United States; ^2^Comparative Medicine Institute, North Carolina State University, Raleigh, NC, United States; ^3^Department of Molecular Biomedical Sciences, College of Veterinary Medicine, North Carolina State University, Raleigh, NC, United States

**Keywords:** tendon, cytokine, ultrafiltration probe, mesenchymal stem cell, licensing

## Abstract

Tendon injury in the horse carries a high morbidity and monetary burden. Despite appropriate therapy, reinjury is estimated to occur in 50–65% of cases. Although intralesional mesenchymal stem cell (MSC) therapy has improved tissue architecture and reinjury rates, the mechanisms by which they promote repair are still being investigated. Additionally, reevaluating our application of MSCs in tendon injury is necessary given recent evidence that suggests MSCs exposed to inflammation (deemed MSC licensing) have an enhanced reparative effect. However, applying MSC therapy in this context is limited by the inadequate quantification of the temporal cytokine profile in tendon injury, which hinders our ability to administer MSCs into an environment that could potentiate their effect. Therefore, the objectives of this study were to define the temporal cytokine microenvironment in a surgically induced model of equine tendon injury using ultrafiltration probes and subsequently evaluate changes in MSC gene and protein expression following *in vitro* inflammatory licensing with cytokines of similar concentration as identified *in vivo*. In our *in vivo* surgically induced tendon injury model, IL-1β and IL-6 were the predominant pro-inflammatory cytokines present in tendon ultrafiltrate where a discrete peak in cytokine concentration occurred within 48 h following injury. Thereafter, MSCs were licensed *in vitro* with IL-1β and IL-6 at a concentration identified from the *in vivo* study; however, only IL-1β induced upregulation of multiple genes beneficial to tendon healing as identified by RNA-sequencing. Specifically, vascular development, ECM synthesis and remodeling, chemokine and growth factor function alteration, and immunomodulation and tissue reparative genes were significantly upregulated. A significant increase in the protein expression of IL-6, VEGF, and PGE2 was confirmed in IL-1β-licensed MSCs compared to naïve MSCs. This study improves our knowledge of the temporal tendon cytokine microenvironment following injury, which could be beneficial for the development and determining optimal timing of administration of regenerative therapies. Furthermore, these data support the need to further study the benefit of MSCs administered within the inflamed tendon microenvironment or exogenously licensed with IL-1β *in vitro* prior to treatment as licensed MSCs could enhance their therapeutic benefit in the healing tendon.

## Introduction

Tendon injury in the horse, most commonly affecting the superficial digital flexor tendon (SDFT), has a high morbidity and monetary burden associated with treatment, rehabilitation, and lost athletic performance (1, 2). Despite appropriate therapy, high reinjury rates are estimated to occur in 50–65% of cases (1–3). In the last 20 years, implementation of mesenchymal stem cell (MSC) therapy has secured a prominent position in treating a variety of musculoskeletal injuries. Specifically, intralesional administration of MSCs in horses has improved SDFT architecture in experimental settings and reduced reinjury rate in clinical cases (3, 4). Despite these improved outcomes following MSC therapy, new insights into MSC biology have created a paradigm shift. It is now believed that the effect of MSCs is not due to local engraftment and differentiation, but instead from secretion of bioactive paracrine factors commonly referred as the secretome (5, 6). Particularly, data have shown that inflammatory molecules stimulate, or license, MSCs to enhance their secretion of reparative and immunomodulatory factors to a greater degree than in their naïve, or unlicensed, state (7–10). While this has translated to increased research in licensed MSCs for other inflammatory diseases, minimal work has examined inflammatory licensed MSCs for the treatment of tendon injuries (11).

Understanding the tissue microenvironment during injury guides our ability to develop new therapies to combat detrimental molecular mechanisms and encourage local reparative factors to improve healing. However, as recently reviewed (12), our knowledge of the temporal cytokine profile following tendon injury is limited due, in part, to the difficulty in obtaining adequate antemortem samples without compromising tendon structure and function. Our current understanding of the tendon microenvironment following injury has relied upon simplified models of injury in cell culture, intermittent post-mortem analyses in terminal animal disease models, or evaluation of human clinical patients with naturally occurring, but non-standardized tendon injuries (13, 14). Therefore, more thoroughly determining the temporal change in the cytokine profile in a standardized tendon injury model would advance our knowledge of the molecular mechanisms that drive healing and disease, which could subsequently advance our ability to develop more effective therapies for tendon injury. Furthermore, examining how this inflammatory microenvironment affects MSCs and their secreted factors in the context of MSC licensing has the potential to advance MSC therapy for tendon injuries.

The objectives of this study were 2-fold: (1) define the *in vivo* temporal cytokine profile of the tendon microenvironment using a novel ultrafiltration probe implanted into a surgically induced tendon lesion in the horse; and (2) determine how the predominant inflammatory cytokines identified in **(author?)** (1) affect the MSC gene and protein expression *in vitro*. We hypothesized that the use of a novel ultrafiltration probe would allow us to define the temporal cytokine profile in a surgically induced model of tendon injury. Further, we hypothesized that licensing of MSCs separately with IL-1β or IL-6 at the concentration identified in the *in vivo* tendon microenvironment would enhance tendon-beneficial gene and protein expression.

## Materials and methods

### Animal use and welfare

Seven Thoroughbred horses (age median 5 years, range 4–13 years; median weight 490 kg, 405–513 kg), free of clinical lameness and palpable or ultrasonographically evident tendon injury were enrolled, and underwent surgical induction of a core lesion of both forelimb SDFTs in an experimental protocol approved by the Institutional Animal Care and Use Committee of North Carolina State University (Protocol #20-389). For the entire study period, horses were housed in an individual 12 × 12 foot concrete stall with rubber floor mats with bedding and provided water and grass hay *ad libitum* in a temperature-controlled facility. Preoperative complete blood count and serum chemistry were performed prior to surgery and weekly thereafter.

### Ultrafiltration probe development and surgical technique

The ultrafiltration probes used were a modified version of a commercially available probe (BASi Research Products, West Layfayette, IN, USA) used for pharmacokinetic and pharmacodynamic (PK/PD) modeling studies. These probes consist of three loops of membrane with a 30 kDa molecular weight cutoff, joined to a single, non-permeable conducting tube (15, 16). Our group, with assistance from BASi Research Products, performed both *ex vivo* and *in vivo* pilot studies to custom design a modified ultrafiltration probe with a slimmer profile consisting of a single loop of membrane with a 100 kDa molecular weight cutoff to ensure collection of larger cytokines like TNF-α (17).

**Figure 1:**
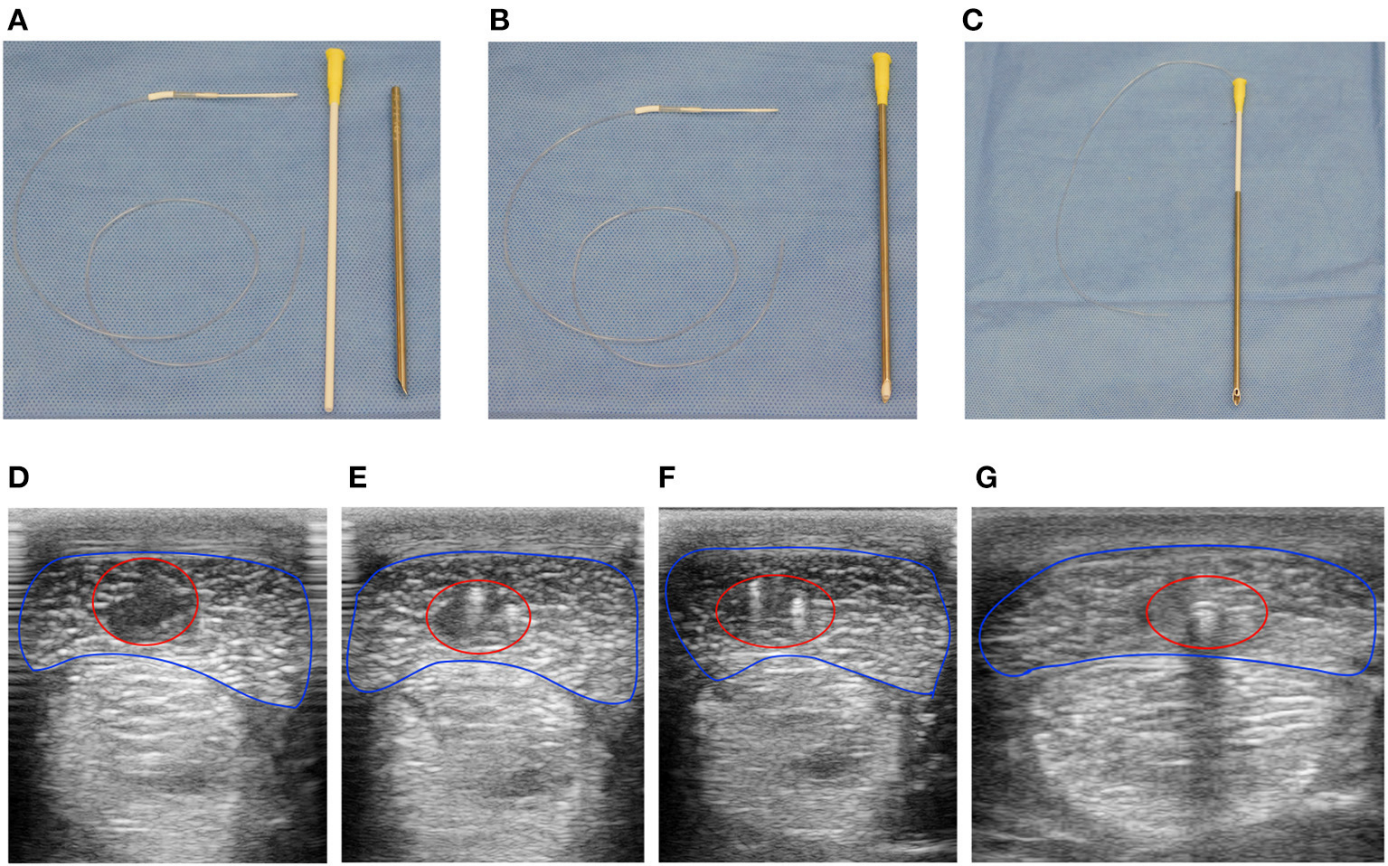
Ultrafiltration probes were easily placed under ultrasound guidance within the center of surgically induced equine SDFT lesions. Instruments required to place ultrafiltration probes within surgically induced lesions of the SDFT. **(A)** Ultrafiltration probe (left), venous catheter (middle), and Basi^®^ introducer needle (right). The venous catheter was threaded normograde into the introducer needle **(B)**, followed by retrograde threading of the ultrafiltration probe into the venous catheter-introducer needle **(C)**. Using ultrasound guidance **(D–G)**, the device facilitated placement of the ultrafiltration probe within tendon lesions (red circle) of the SDFT (blue outline) following surgical induction. At the most proximal extent **(D)** a large hypoechoic lesion with loss of tendon fiber is visualized. Moving distally, the two fibers of the ultrafiltration probe loop can be visualized as hyperechoic foci within the hypoechoic lesion **(E,F)**. Finally, acoustic shadowing due to gas within the lumen of the conducting tubing **(G)** was observed at the most distal extent of the tendon lesion proximal to tube exit from the tendon and skin.

Horses were anesthetized, moved to an operating theater, and maintained with general inhalant anesthesia. Core lesions were created in the extrasynovial portion of both forelimb SDFTs of seven horses using a previously published method shown to induce consistent injury with a similar ultrasonographic and histologic progression encountered in naturally occurring equine SDFT injury (18). Briefly, following aseptic preparation, local anesthesia with bupivacaine liposome injectable suspension (Nocita^®^, Elanco Animal Health, Inc., Greenfield, IN) of the medial and lateral palmar metacarpal nerves and medial and lateral metacarpal nerves was performed immediately distal to the head of the second and fourth metacarpal bones and tendon lesions induced under ultrasonographic guidance as previously described. Thereafter, a custom-designed 100 kDa ultrafiltration probe (BASi Research Products, West Lafayette, IN, USA) was threaded retrograde through a 12-gauge venous catheter (Zoetis, Inc., Kalamazoo, MI, USA) to seat the probe entirely within the lumen (Figures 1A,B). The probe-venous catheter was then inserted within the lumen of an ultrafiltration probe introducer needle (BASi Research Products, West Lafayette, IN, USA, Figure 1C). Using this method, the function of the introducer needle was placement of the ultrafiltration probe within the lesion while the venous catheter provided support at the probe base to ensure it was maintained within the tendon when the introducer needle was removed. Following placement of ultrafiltration probes in six horses, the catheter and introducer needle were removed, and probe location confirmed with ultrasonography (Figures 1D–G). A single horse underwent creation of bilateral SDFT lesions without placement of ultrafiltration probes to serve as control. Skin incisions of all horses were closed with 2-0 polypropylene in a simple interrupted pattern, the limbs bandaged standardly, and horses recovered from general inhalant anesthesia.

Horses received prophylactic gentamicin sulfate (6.6 mg/kg, q 24 h, IV) and penicillin G potassium (22,000 IU/kg, q 6 h, IV) prior to surgery and for 3 days after. Postoperative analgesia was provided by morphine sulfate (0.05–0.1 mg/kg, q 12–24 h, IM) for 2–3 days depending on comfort. Non-steroidal anti-inflammatory drugs (NSAIDs) were purposefully avoided to prevent alteration of the tendon cytokine profile. Following recovery from general anesthesia, each limb was unbandaged, a sterile evacuated tube containing protease inhibitors (BD, Franklin Lakes, NJ) attached to the needle and conducting tube system of the ultrafiltration probe, and the limb rebandaged. Tendon ultrafiltrate was collected from evacuated tubes within the first 3–5 h following surgery and a new tube replaced and changed every 12 h thereafter. Samples were aliquoted into cryovials immediately following collection and stored at −80°C until analysis within 4 months following collection. Ultrasound-guided percutaneous aspiration of lesions of the single control horse was attempted on day one, three, seven, and ten postoperatively and samples aliquoted and stored as with tendon ultrafiltrate. Horses were hand walked for five (week one), ten (week two), and fifteen (week three) minutes twice daily until the conclusion of the study. At 21 days following surgery, horses were sedated and euthanized with an intravenous overdose of sodium pentobarbital. Tendon from a representative limb was collected for histopathology in two horses.

### Cytokine characterization

Cytokine concentration for equine-specific interleukin-1β (IL-1β),−6,−8,−10, fibroblast growth factor-2 (FGF-2), tumor necrosis factor-α (TNF-α), interferon-γ (IFN-γ), interferon inducible protein-10 (IP-10), transforming growth factor-β1 (TGF-β1),−2, and−3 were measured in tendon ultrafiltrate with a fluorescent bead-based multiplex assay (MILLIPLEX, Equine Multiplex Assay, MilleporeSigma, MA, USA) and performed according to manufacturer's instructions. For the multiplex assay, concentrations measured below the lower limit of quantification (LLoQ) were assigned a value of 0.1 of the LLoQ. Prostaglandin E2 (PGE2) was determined using a colorimetric competitive ELISA according to manufacturer's instructions (Enzo Life Sciences, Inc., Farmingdale, NY, USA). All samples were analyzed in duplicate. Tendon ultrafiltrate from later timepoints was pooled for TGF-β and PGE2 analysis due to small sample volume.

### RNA sequencing of licensed MSCs

Banked equine bone marrow-derived MSCs (*n* = 6, donor horse age median 9 years, range 6–12 years) were utilized for this study and were isolated as previously described (19). MSCs were thawed and seeded in complete MSC media (19) containing 10% fetal bovine serum (FBS) and when cultures reached 80% confluency, MSCs were passaged with Accutase cell-dissociation solution (Innovative Cell Technologies) and 0.35 × 10^6^ cells plated on 100 mm culture plates for RNA sequencing experiments. MSCs were passaged 2–3 times prior to use. Twenty-four hours following passage and adherence, cells were licensed by adding complete MSC media containing 2 ng/ml recombinant human IL-1β (R&D Systems) or 2 ng/ml recombinant human IL-6 (BioLegend) for 72 h with media exchanged at 48 h. After 72 h, cells were washed and lifted in preparation for RNA-sequencing.

Total RNA was extracted from paired P3 or P4 naïve, IL-1β licensed, and IL-6 licensed MSCs from six donor horses using the RNeasy Mini kit (Qiagen). Libraries were generated and poly(A) enriched using 1 μg of RNA as input. Indexed samples were sequenced using a 150 base pair paired-end protocol on a HiSeq 2500 (Illumina) according to the manufacturer's protocol. Sequence reads were trimmed to remove possible adaptor sequences and nucleotides with poor quality using Trimmomatic v.0.36. The trimmed reads were mapped to the EquCab 3.0 reference genome available on ENSEMBL using the STAR aligner v2.5.2b. Unique gene hit counts were calculated using featureCounts from the Subread package v1.5.2. Using DESeq2, a comparison of gene expression between the naive and IL-1β or IL-6 licensed MSCs was performed. The Wald test was used to generate *p*-values and log2 fold changes. Genes with an adjusted *p*-value of <0.05 and log2 fold change >1 were determined to be differentially expressed for each comparison. The quantification and poly(A) selection of mRNA, library preparation, sequencing, and bioinformatics were outsourced to GENEWIZ, Inc.

### MSC protein expression

Supernatants from paired naïve and licensed MSCs (*n* = 4, donor horse age median 17 years, range 12–23 years) were collected, spun at 500*xg* for 10 min to remove cellular debris, and stored at −80°C until analysis. Protein expression of IL-1β licensed MSCs were evaluated with ELISA assays for equine IL-6 (R&D Systems, Minneapolis, MN, USA), human PGE2 (Enzo Biochem, Inc., Farmingdale, NY, USA), and equine VEGF (Kingfisher Biotech, Saint Paul, MN, USA) and performed per manufacturer's instructions. For PGE2 analysis, supernatant from IL-1β licensed MSCs was diluted 1:100 in reagent diluent.

### Statistical analysis

Tendon ultrafiltrate volumes were assessed for normal distribution by Shapiro-Wilk test. Descriptive analysis was performed for both tendon ultrafiltrate volume and cytokine concentration. Normally distributed data was reported as mean ± standard deviation while non-parametric data reported with median and interquartile range. A one-tailed paired *t*-test was used to examine difference in protein expression for ELISA assays. All analysis and figure preparation were performed using GraphPad Prism (GraphPad Software Inc; La Jolla, CA, USA). The functional enrichment analysis of RNA-sequencing data was performed using g:Profiler (version e104_eg51_p15_3922dba) with g:SCS multiple testing correction method applying significance threshold of 0.05.

## Results

### Animals and surgical implantation

An ultrafiltration probe was implanted in each core lesion of six horses. A single horse did not have ultrafiltration probes implanted to serve as control. All horses recovered from general anesthesia without incident and remained comfortable through the entirety of the 21-day study. Complete blood count and serum chemistry for all horses were unremarkable for the duration of the study. Ultrafiltration probes were easy to place within surgically induced lesions; however, maintenance of ultrafiltrate flow following surgery was more difficult as early ultrafiltrate had the tendency to slow or fully obstruct flow at the lumen of the needle connecting the conducting tubing to the evacuated tubes. However, once a new needle was exchanged, collection of tendon ultrafiltrate into the evacuated tubes resumed.

### Cytokines in tendon microenvironment

Tendon ultrafiltrate was collected from the majority of ultrafiltration probes throughout the 21-day period; however, volume and frequency of collection varied between horses and between limbs of the same horse throughout (Supplementary Table 1). No significant difference was found in total ultrafiltrate volume collected over the 21-day period between individual horses and was normally distributed (4.411 ± 2.325 ml; *p* = 0.944).

**Figure 2:**
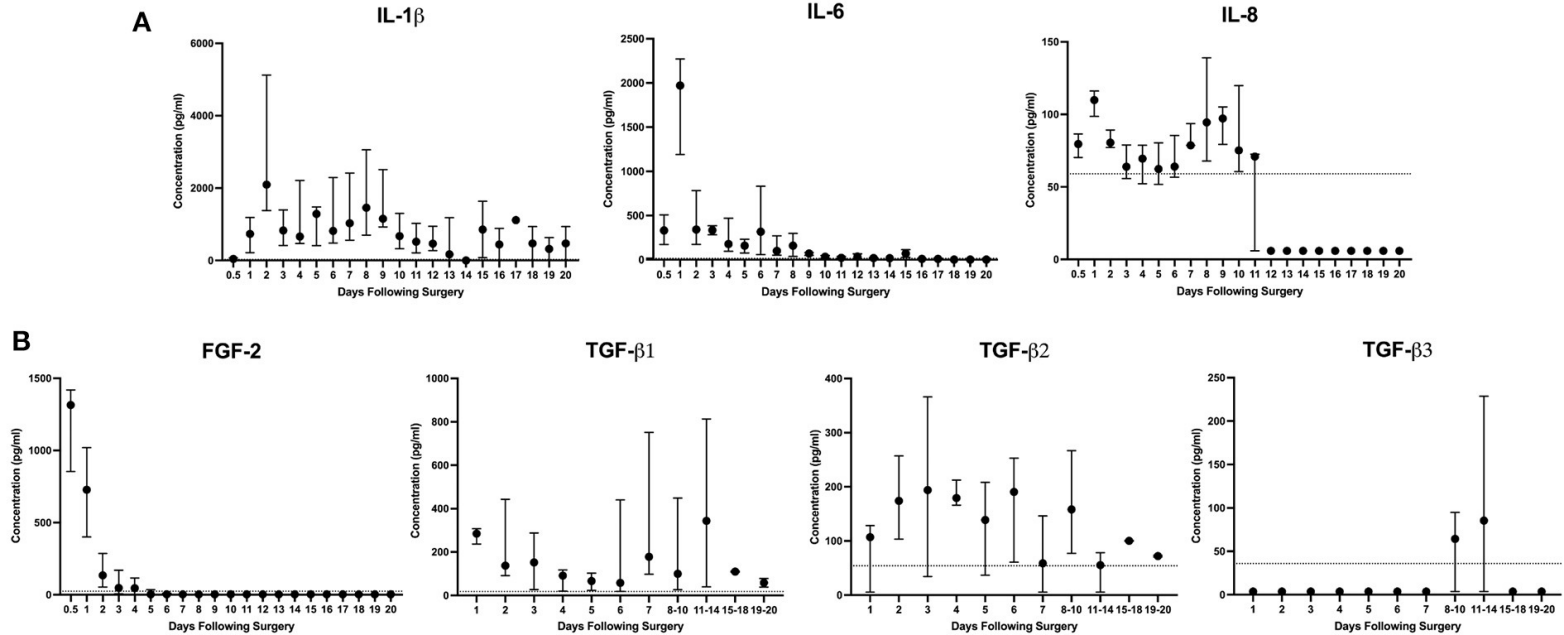
IL-1β and IL-6 are the predominant inflammatory cytokines present following surgically induced equine SDFT injury. Descriptive report of **(A)** inflammatory cytokines and **(B)** growth factors FGF-2 and TGF-β1,−2, and−3 in tendon ultrafiltrate were measured by ELISA following bilateral surgically induced SDFT injury of the forelimb over 21 days in 6 horses. Due to insufficient volume, samples from days 8 onward were grouped for TGF-β isoform analysis. When present, the dashed line at the y-axis represents the lower limit of quantification (LLoQ) for that cytokine or growth factor as determined by the assay manufacturer (MilliporeSigma, MA, USA). Concentrations measured below the LLoQ were assigned a value of 0.1 of the LLoQ.

Overall, pro-inflammatory cytokines peaked within the first 24–48 h (Figure 2A). IL-1β at 48 h (median 2,097 pg/ml, 668–5,505 pg/ml), IL-6 at 24 h (median 1,971 pg/ml, 1,114–2,475 pg/ml), and IL-8 at 24 h (median 110 pg/ml, 96–120 pg/ml). Quantifiable levels of IFN-γ, TNF-α, IP-10, IL-10 were not detected. More acutely, the highest levels of FGF-2 were identified within the first sampling period before 12 h (median 1,315 pg/ml, 605–1,423 pg/ml, Figure 2B). TGF-β isoforms were detected at varying times throughout the 21-day period (Figure 2B). TGF-β1 was measured in tendon ultrafiltrate throughout with a biphasic peak at 24 h (median 285 pg/ml, 221–314 pg/ml) and days 11–14 (median 344 pg/ml, 23.0–885 pg/ml). TGF-β2 levels peaked at day 3 (194 pg/ml, 5.4–399 pg/ml) and remained similar through the first week before declining over the final 10 days of the study. Finally, TGF-β3 was below the lower limit of detection except for on days 8–10 and at its peak at days 11–14 (median 85.4 pg/ml, 3.60–249 pg/ml). The concentration of PGE2 was initially measured in two horses and was below quantifiable limits in samples prior to day 15–18. Due to concerns about volume remaining for other assays, further characterization of PGE2 was abandoned. Only small volumes of hemorrhagic sample were obtained during attempts at ultrasound-guided percutaneous aspiration of the control horse lesions. Most cytokines measured from samples were below quantifiable level. This along with concern regarding peripheral blood contamination prevented analysis of samples from the single control horse.

Postmortem histopathology (hematoxylin and eosin) of representative tendons revealed presence of mildly reactive fibroblasts with extracellular matrix deposition within the tendon and surrounding ultrafiltration probe fibers (Supplementary Figure 1). Due to lack of indicators of chronic inflammation such as adjacent leukocyte infiltration, ultrafiltration probe fibers were determined to be inert as consistent with previous literature on the use of ultrafiltration probes in other locations within the body (15, 20, 21).

### IL-1β licensed MSCs, but not IL-6 licensed, have enhanced reparative gene expression

**Figure 3:**
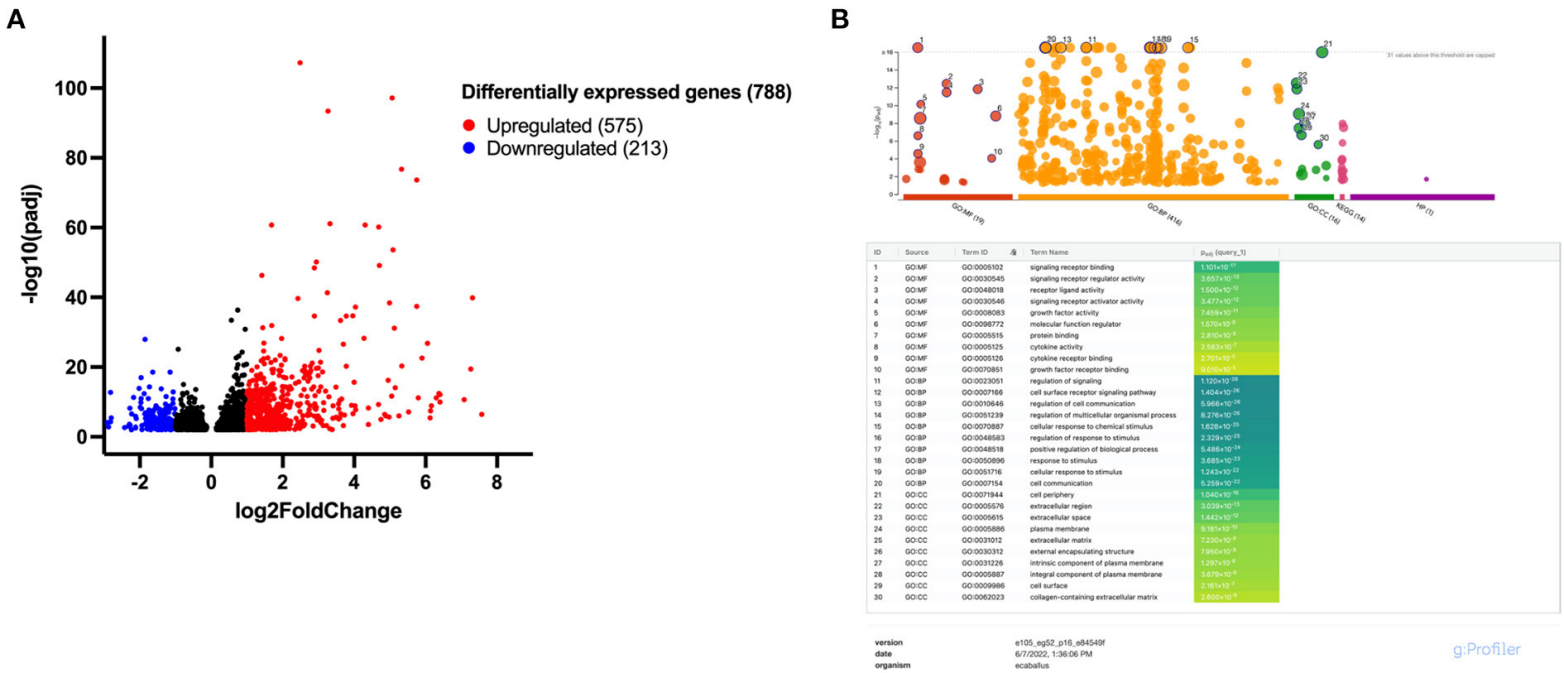
Approximately 788 differentially expressed genes (DEGs) were identified following IL-1β licensing of equine MSCs with overrepresented terms including growth factor and cytokine activity and extracellular matrix-associated functions. **(A)** Volcano plot for differential expression analysis depicting the log_2_ fold change and -log_10_ (adjusted *p* value) for gene transcripts in IL-1β licensed MSCs detected by RNA-sequencing. Significantly upregulated DEGs are presented in red and significantly downregulated DEGs in blue with absolute fold change >1.0 and a *p*-value < 0.01. **(B)** Manhattan plots from g:Profiler illustrating GO term enrichment analysis of DEGs of MSCs following IL-1β licensing. The top 10 enriched pathways for each GO term (MF, molecular function; BP, biological process; CC, cellular component) are presented.

Following IL-1β licensing of MSCs, a total of 788 significant differentially expressed genes (DEGs) were identified with 575 genes upregulated and 213 genes downregulated (Figure 3A). Those genes with the largest increase in expression included: NOS2, a key regulator of nitric oxide production, a reactive free radical involved in a variety of biologic processes (22); CCL20, an inflammatory chemokine that binds exclusively to CCR6 receptors commonly found in cells of the immune system (23); IL6, a pleotropic cytokine associated with inflammation, tissue healing, metabolism, and lymphocyte development (24); and FOXN4, a member of the forkhead box/winged-helix transcription superfamily that determines cell fate in neural and non-neural tissues (25). Genes with the largest downregulation included LOC106780963, an uncharacterized gene from chromosome 9; LMCD1, a regulator of MSC osteogenic differentiation (26); PGFS, an enzyme responsible for prostaglandin F production which causes bronchial and arterial smooth muscle contraction (27); and MGP, a vitamin K-dependent protein associated with vascular calcification (28). Gene ontology (GO) enrichment analysis identified the top overrepresented terms in the entire DEG set. The top ten molecular functions (MF), biological processes (BP), and cellular components (CC) for these data are listed (Figure 3B). Overrepresented terms identified by GO analysis included growth factor activity, cytokine activity, cell communication, extracellular matrix, and collagen-containing extracellular matrix among others (Figure 3B). For the top 100 upregulated genes, all significant GO terms for MF, BP, CC, KEGG pathways, and human phenotype ontology (HP) are shown in Supplementary Figure 2.

**Figure 4:**
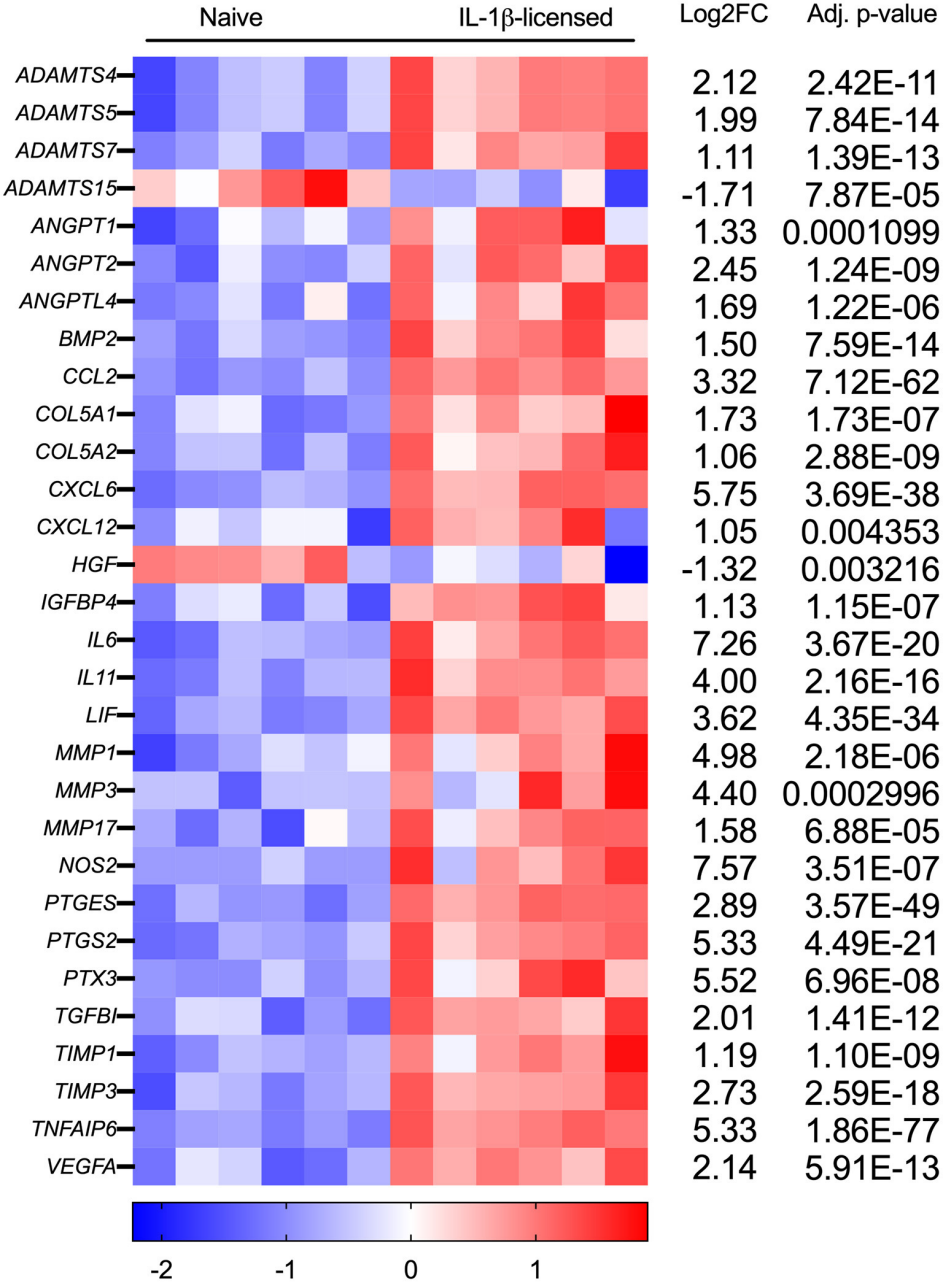
Tendon-relevant genes are significantly differentially regulated in equine MSCs following IL-1β licensing. Heat map representation using log2fold change of select significantly differentially expressed genes (DEGs) from RNA sequencing analysis following IL-1β licensing (*n* = 6 horses). Compared to naïve MSCs, gene downregulation is noted in blue and gene upregulation in red.

Of the 788 DEGs, multiple genes were identified that could be relevant—whether beneficial or detrimental—to the healing tendon (Figure 4; Table 1). Multiple differentially regulated genes were identified, that if translated to their downstream proteins, could have tendon-relevant effects (Table 1) through tendon or MSC-specific function including those associated with vascular development (ANGPT1/2, ANGTPL4, CXCL6, VEGF-A, VEGF-C), synthesis and remodeling of the tendon ECM (ADAMTS4/5, COL5A1/A2, IL6, MMP1/3/17, NOS2, TIMP1/3), chemokine and growth factor function alteration (MMP1/3), and immunomodulation and tissue reparative effects (IL6, NOS2, PTGES, PTGS2). Following MSC licensing with IL-6, EGR3 and FOSB were the lone DEGs identified. Neither was considered relevant to MSC function in the context of tendon development or healing.

**Table 1:** Function of downstream proteins produced from DEGs identified from IL-1β licensed MSCs that could impart tendon-relevant effects.

**Gene**	**Log2 fold change**	***p*-value**	**Function of downstream protein**
ADAMTS4	2.119087765	3.89E-13	Inhibited by TIMP3 (29), cleave Glu-X bond of aggrecan core protein (30)
ADAMTS5	1.986039748	8.24E-16	Cleave Glu-X bond of aggrecan core protein (30)
ADAMTS7	1.112947643	1.53E-15	Smooth muscle cell migration and COMP digestion (30)
ADAMTS15	−1.705114295	5.43E-06	Cleave Glu-X bond of aggrecan core protein (30)
ANGPT1	1.331215264	7.98E-06	Support vascular maintenance and homeostasis, promotes self-renewal of adult muscle stem cells (31)
ANGPT2	2.450315564	2.59E-11	Can agonize or antagonize angiopoietin-1 and dependent on concurrent presence of VEGF (31, 32)
ANGPTL4	1.694800283	4.99E-08	Enhances *in vitro* tenocyte migration, regulates tenocyte cell cycle genes (33), angiogenesis (34)
BMP2	1.504661232	7.84E-16	Exogenous administration induces expression of collagen type I in human tenocytes (35) and in equine tenocytes overexpressing BMP2 (36), but can induce mineralization in both equine tenocytes/MSCs overexpressing BMP2 (36)
CCL2	3.321170222	2.92E-65	Associated with GDF5-induced human MSC tenogenic differentiation (37), in conjunction with CXCL12, induces MO polarization and secretion of IL-10 (38)
COL5A1	1.729683994	5.68E-09	Encodes one of the three alpha chain to assemble collagen type V; forms heterofibrils with collagen type I and II (39)
COL5A2	1.056264925	6.59E-11	Encodes one of the three alpha chain to assemble collagen type V; forms heterofibrils with collagen type I and II (39)
CXCL6	5.752098101	4.79E-41	Ability to indue angiogenesis (40), induces MSC secretion of pro-angiogenic genes (41)
CXCL12	1.051902676	0.000612448	In conjunction with CCL2, induces MO polarization and secretion of IL-10 (38)
HGF	−1.324646103	0.000422508	Inhibits TGFβ-1-induced myofibroblast differentiation of rat tendon fibroblasts (42)
IGFBP4	1.133446579	3.67E-09	Secreted by senescent MSCs and induces senescence in previously unaffected MSCs (43)
IL6	7.262226972	1.68E-22	Increased peritendinous COL1 terminal telopeptide concentrations (44), induced through *in vitro* mechanical stretching of healthy human tendon fibroblasts (45), increased gene expression in chronic human tendinopathy (46)
IL11	4.001668021	1.62E-18	Driver of tissue fibrosis (47), induces TIMP production in articular chondrocytes/synovial fibroblasts (48)
LIF	3.615225809	7.72E-37	IL-6 cytokine family member responsible for induction of JAK-STAT signaling pathway
MMP1	4.975771726	9.42E-08	Secreted-type collagenase with ability to bind native, triple helical collagen type I-III and CTGF (30), selectively degrades CTGF from the CTGF-VEGF complex and restores VEGF angiogenic activity (49), chemokine (CCL2, CXCL12 others) inhibition (50)
MMP3	4.400692659	2.56E-05	Secreted-type stromelysin that selectively degrades CTGF from the CTGF-VEGF complex and restores VEGF angiogenic activity (49), chemokine (CCL2, CXCL12 others) inhibition (50)
MMP17	1.579842693	4.66E-06	Membrane-anchored MMP associated with pericellular proteolysis (30)
NOS2^*^	7.569179419	1.24E-08	Increased tenocyte collagen synthesis (51), reduction in tendon CSA and load-to-failure when inhibited (52, 53)
PTGES	2.88704628	3.17E-52	Enzyme responsible for terminal step of transformation of COX-derived PGH2 into PGE2 (54)
PTGS2^*^	5.3337729	1.81E-23	Primary enzyme responsible for controlling PGE2 synthesis in response to inflammation (55)
PTX3	5.521333296	2.16E-09	Anti-inflammatory effects when bound to hyaluronic acid-heavy chain complex induced by TSG-6 (56)
TGFBI	2.011919175	1.82E-14	Negative regulator of TLR-induced inflammation (57), chondroprotective (58)
TIMP1	1.190793601	2.28E-11	Variable ability to inhibit various MMPs and ADAMs (30), regulation of cell migration (59)
TIMP3	2.726335861	1.56E-20	Inhibit ADAMTS-4,−5, various ADAMs (including ADAM17/TACE), and pro-MMP-9 (cleaves denatured collagen) (30), regulate cytokine signaling and inflammatory cell adhesion (50), inhibits angiogenesis through VEGFR2 binding (60)
TNFAIP6	5.325227457	5.08E-81	Results in TSG-6 responsible for anti-inflammatory and tissue protective/reparative effects mediated through TSG-6 binding of matrix proteins and chemokines, essential for TGF-β1-induced fibroblast to myofibroblast differentiation, and inhibits BMP-2 driven osteoblast differentiation of MSCs (56)
VEGFA	2.144734795	7.27E-15	Induce hemangiogenesis (61); variable effects identified based on model, VEGF isoform, and timing of exogenous delivery (62, 63); stimulation of MMPs and inhibition of TIMPs (64, 65)

Gene name, log2fold change, and p-value from RNA sequencing data of MSCs following IL-1β licensing with literature to support tendon-relevant function through tendon or MSC-specific function.

ADAMTS, a disintegrin and metalloproteinase with thrombospondin motif; ANGPT, angiopoietin; ANGPTL, angiopoietin like; BMP, bone morphogenetic protein; CCL, C-C motif chemokine ligand; COL, collagen; COMP, cartilage oligomeric matrix protein; COX, cyclooxygenase; CSA, cross sectional area; CTGF, connective tissue growth factor; CXCL, C-X-C motif chemokine ligand; HGF, hepatocyte growth factor; IGFBP, insulin like growth factor binding protein; IL, interleukin; LIF, leukemia inhibitory factor; MMP, matrix metalloproteinase; MO, macrophage; MSC, mesenchymal stem cell; NOS, nitric oxide synthase; PGE2, prostaglandin E2; PGH2, prostaglandin H2; PTGES, prostaglandin E synthase; PTGS, prostaglandin-endoperoxide synthase; PTX, pentraxin; TGFβ, transforming growth factor beta; TGFBI, transforming growth factor beta induced; TIMP, tissue inhibitor of metalloproteinase; TACE, TNF-α converting enzyme; TNFAIP, TNF alpha induced protein; TSG-6, TNF-stimulated gene-6; VEGFA, vascular endothelial growth factor A.

* Also known as: NOS2 = iNOS, inducible NOS; PTGS2 = COX-2 (cyclooxygenase-2).

### IL-1β licensed MSCs secrete significantly more IL-6, PGE2, and VEGF than naïve MSCs

**Figure 5:**
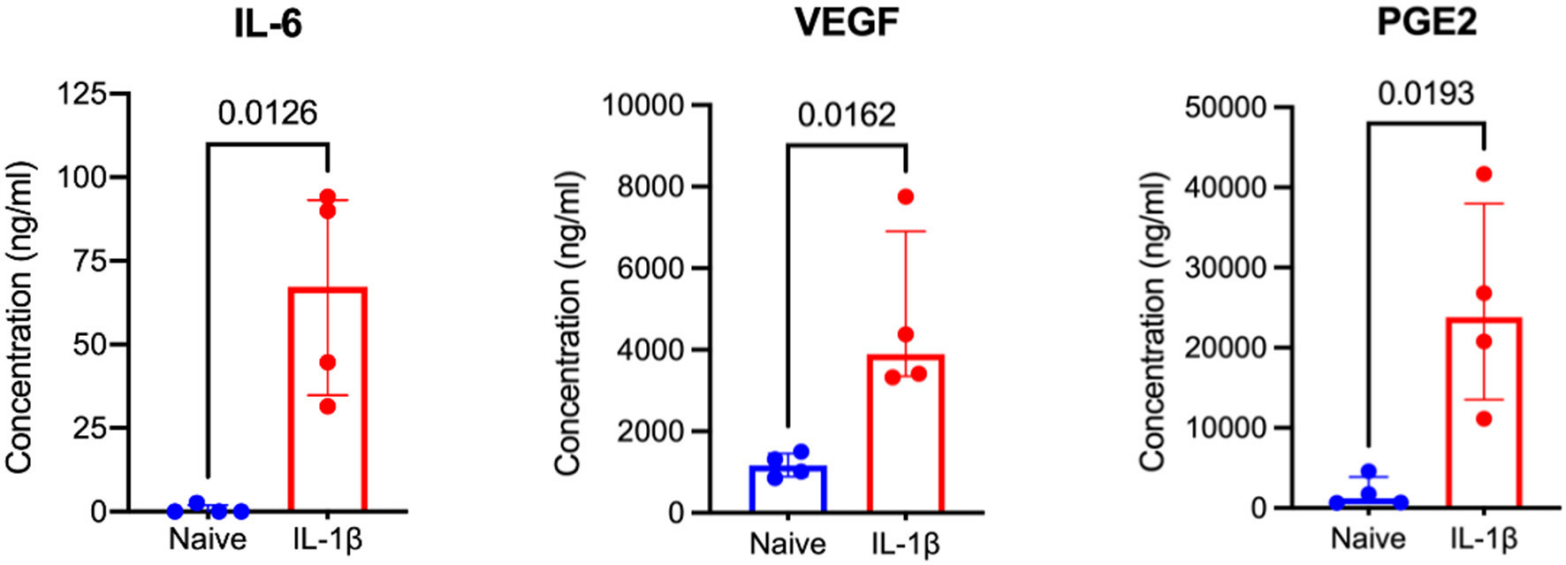
IL-1β licensed equine MSCs produce significantly more IL-6, VEGF, and PGE2 than naïve MSCs. From RNA sequencing data, three proteins known to have tendon-relevant effects were selected to confirm downstream protein expression. Analysis of MSC cell culture supernatants by ELISA assay confirmed increased protein expression of IL-6, VEGF, and PGE2 following IL-1β licensing compared to naïve MSC controls. A one-tailed paired *t*-test was used to examine difference in protein expression between groups.

Three DEGs in IL-1β-licensed MSCs identified in RNA-seq data were chosen due to their known relevance to tendon healing, to confirm downstream protein expression. Cell culture supernatant obtained from IL-1β licensed MSCs produced significantly more IL-6 (*p* = 0.0126), PGE2 (*p* = 0.0193), and VEGF (*p* = 0.0162) compared to their paired naïve controls (Figure 5). IL-6 is a pleotropic cytokine present following tendon injury and regulates leukocyte infiltration and controls duration of pro-inflammatory mediator release (12, 66). Prostaglandin E2 is a primary mediator of equine MSC immunomodulation, is present following tendon injury, and likely plays a critical, concentration-dependent role in both the reparative response as well as the development of chronic injury (67, 68). VEGF, present following tendon loading and tenocyte mechanical stimulation, appears to improve strength of healing tendon when administered exogenously (69). These data support that IL-1β licensing is inducing changes in gene and protein expression in MSCs and this phenotype could provide tendon-relevant effects.

## Discussion

This is the first study of its kind to define the temporal cytokine profile following surgically induced tendon injury. A novel method of implantation of an ultrafiltration probe within the center of the equine SDFT was implemented where it was determined that inflammation was predominated by IL-1β and IL-6. Additionally, concentrations of both cytokines peaked at ~2,000 pg/ml (2 ng/ml) within 48 h following injury. When MSCs were subsequently licensed with a similar concentration of exogenous recombinant human IL-1β, licensed MSCs upregulated expression of multiple genes that could impart benefit to the healing tendon. Further, downstream expression for three select genes were supported by significantly higher protein concentrations measured in culture supernatant compared to naïve MSCs. These data further improve our understanding of the tendon microenvironment following injury and provide evidence that IL-1β licensed MSCs could provide enhanced therapeutic benefit compared to their naïve counterparts.

Elucidating the temporal cytokine profile in tendon following injury is critical to guide development of novel therapies that aim to return the biochemical composition and biomechanical strength of healing tendon to its native state. Attempts to characterize the interplay of cytokines in the tendon microenvironment in both health and disease have employed cell culture, tissue biopsy, and subcutaneous microdialysis as recently review by Ellis et al. (12). However, the inability to repeatedly sample the injured tendon microenvironment in conjunction with inconsistent standardization of samples from naturally occurring disease limit our understanding of the temporal change that occurs following injury (13, 14). To rectify this, our group was able to sample the tendon microenvironment twice daily for 21 days in a surgically induced model using a novel method that employed ultrafiltration probes. The distinct advantages of this technique include standardization of tendon lesions, implementation of a relevant large animal model of tendon injury, and daily temporal characterization of the tendon microenvironment that would otherwise require large numbers of terminal animal studies (18, 70–72). We demonstrate that ultrafiltration probes were effective in facilitating characterization of the cytokine milieu in tendon injury. Additionally, lack of histopathologic evidence of foreign body reaction (73) within examined tendons indicate the cytokines at their measured concentration were an accurate reflection of the tendon microenvironment following injury. While we did not attempt to elucidate the cell-specific source of these cytokines, *in vitro* studies support this milieu is likely secreted by a combination of local and infiltrating cells including tendon fibroblasts, tendon stem/progenitor cells, and immune cells (14, 74, 75). The authors recognize that although the cytokine quantification herein is limited to the analytes present in the commercial kits and does not represent all analytes involved in the molecular cascade, the novel method employed to characterize the tendon microenvironment is an essential first step and establishes a new technique to collect further data on the microenvironment following injury and in response to therapy. Despite the associated bias when selecting cytokines to analyze tendon ultrafiltrate, published literature of the injured tendon microenvironment (12, 13, 76) was used to support selection of the most relevant cytokines in our custom multiplex within species-specific assay constraints.

While the therapeutic effect of MSCs was previously attributed to local engraftment and cell differentiation, it is now understood that MSC functions include their ability to stimulate angiogenesis, apoptosis, immunomodulation, and recruitment and stimulation of systemic and endogenous progenitor cells through secretion of paracrine mediators (77, 78). Interestingly, these functions can be dramatically altered when naive MSCs are administered within an injured tissue microenvironment or following exogenous exposure to pro-inflammatory cytokines, deemed MSC licensing (5, 77). In experimental settings, administration of MCSs licensed with inflammatory cytokines or molecules such as bacterial lipopolysaccharide (LPS) can ameliorate pathologic change in the colon (79), improve corneal allograft survival (8), and improve cardiac fibrosis following cardiac ischemia (80). Despite these benefits in various body systems, only two studies have been published that implement MSC licensing and their effect on tendon tissue. Positive outcomes were noted, respectively, in tenocyte gene expression and tendon healing in projects that examined the *in vitro* effect of licensing MSCs with pioglitazone, a hypoglycemic agent, on tenocytes (81) and TNF-α licensed MSCs in a PLG scaffold in a rat Achilles tendon defect (11). From the *in vivo* tendon microenvironment cytokine data, IL-1β and IL-6 were chosen to license MSCs to evaluate effects on gene and protein expression while IL-8 was avoided where it was present at low levels in the tendon microenvironment. Further, from RNA-seq data, naïve equine MSCs were not determined to express IL-8 receptors CXCR1 and−2. Although we understand the effect of IL-1β on MCS gene and protein expression, the influence of IL-6 is less understood (82, 83). Additionally, even less is understood of equine MSCs response following exposure to inflammation with what we do know based on proteomic analysis after IL-1β treatment during chondrogenic differentiation (84). As this is the first report of transcriptome-wide changes in equine MSCs following exposure to inflammatory cytokines, these data presented advance our understanding of MSC biology and will serve as a foundation for future work studying the therapeutic benefit of licensed MSCs.

While increased expression of many genes following IL-1β licensing of MSCs could be beneficial to the healing tendon, it would be remiss to ignore others that conversely could be detrimental. It plausible that IL-1β licensed MCS secretion of IL-11 (tissue fibrosis), reduction in HGF (inhibitor of myofibroblast differentiation), or excessive PGE2 production could be unwanted side effects of treatment. What is poorly understood at this time is how this IL-1β licensed MSC secretome would affect the tendon microenvironment and whether certain molecules impart benefit and trump detrimental effects of others. Assessing the effect of the IL-1β licensed MSC secretome on tenocytes is a critical next step and a current focus of our laboratory. Finally, while genes expanded upon herein were discussed due to their known association with tenocyte and/or MSC function or their role in tendon healing, the authors recognize that many of the other differentially regulated genes could greatly impact the tendon microenvironment if IL-1β licensed MSCs were administered *in vivo*. However, discussion of these genes was omitted as they lack experimental evidence for their role in tendon development and healing.

Our interest is in improving therapy for equine tendon injuries, however, the utility of the horse as a comparative and preclinical model for developing translational therapies for human tendon injury is promising. Human tendon injuries account for more than 32 million musculoskeletal injuries a year in the United States with an increasing incidence and health care-associated cost due to an aging population pursuing more athletic activity (85–87). Despite appropriate therapy, continued high postoperative reinjury rates have shifted focus toward regenerative medicine approaches to improve outcomes (88, 89). However, the lack of randomized, blinded clinical trials combined with tepid results in case series have prevented support for their continued use (90, 91). While no animal model perfectly recapitulates human tendon injury, equine SDFT injury is an accepted comparative model for exercise-induced Achilles injury in humans (72). Additionally, a recent report suggests equine tenocytes have a tendon marker, collagen expression, and ECM gene repertoire most similar to humans (92). With this in mind, data presented herein could be the first step in enhancing MSC therapy for equine tendon injuries while providing the foundation for future translational studies.

Limitations of this study include inconsistent ultrafiltrate collection between limbs of the same horse and between individual horses along with wide individual horse variation in tendon ultrafiltrate cytokine concentration. It is also likely that the cytokine profile described in the context of this acute injury model does not represent the cytokine profile of chronic tendon injury. Characterization of the later and chronic stages of tendon injury are still warranted and may shed light on the potential therapeutic benefit of licensed MSCs for such injury stages. Despite significant upregulation of multiple MSC genes and downstream proteins that could be beneficial to the healing tendon, it is unknown whether greater expression or these bioactive factors all in combination would truly enhance repair and requires further examination. Finally, the authors recognize that while licensing of cells with a single cytokine does appear to improve the reparative gene and protein expression of MSCs, this does not represent all the cytokines, chemokines, and growth factors they would experience in an *in vivo* system and could therefore drastically alter their transcriptome compared to *in vitro* assays.

In conclusion, this is the first study to quantify the temporal cytokine profile over 21 days in an equine model of surgically induced tendon injury. Our results indicate that the tendon microenvironment within the first 48 h following injury contains a concentration of IL-1β that beneficially modulates MSC gene and protein expression and then rapidly resolves. Because current culture expansion techniques require weeks to prepare both newly isolated and banked MSCs, a situation would rarely arise allowing a veterinarian to administer cells into an actively inflamed tendon as a method to improve MSC therapeutic potential. Therefore, an alternative approach could include *in vitro* licensing of MSCs prior to treatment to mimic exposure to this environment. However, before it can be recommended to implement IL-1β licensed MSCs in clinical cases of equine tendon injury, further studies are required examining both their effect on tenocytes in culture as well as in preclinical *in vivo* equine studies if *in vitro* experiments support their use. Overall, data generated in this study greatly expand our knowledge of the cytokine microenvironment during tendon injury and support the continued investigation of IL-1β licensed MSCs as a potential therapeutic.

## Data availability statement

The data presented in the study are deposited in the NCBI GEO repository, accession number GSE208569, and can be found at https://www.ncbi.nlm.nih.gov/geo/query/acc.cgi?acc=GSE208569.

## Ethics statement

The animal study was reviewed and approved by North Carolina State University Institutional Animal Care and Use Committee.

## Author contributions

DK, AB, KM, JG, and LS contributed to study conception and design. DK and AB performed statistical analyses. DK drafted the manuscript with all authors contributing edits. All authors contributed to acquisition of data and interpretation of data. All authors read and approved the final manuscript.

## Funding

This work was supported by the Grayson-Jockey Club Research Foundation (LS) and the Fund for Orthopedic Research in honor of Gus and Equine athletes (F.O.R.G.E; LS) with stipend support from the National Institutes of Health grants T32OD011130 (DK), K01OD027037 (AB), and T35OD011070 (IE).

## Conflict of interest

The authors declare that the research was conducted in the absence of any commercial or financial relationships that could be construed as a potential conflict of interest.

## Publisher's note

All claims expressed in this article are solely those of the authors and do not necessarily represent those of their affiliated organizations, or those of the publisher, the editors and the reviewers. Any product that may be evaluated in this article, or claim that may be made by its manufacturer, is not guaranteed or endorsed by the publisher.
