# Supplementary material for: Interleukin-1β in tendon injury enhances reparative gene and protein expression in mesenchymal stem cells
Source: Front Vet Sci. 2022 Aug 11;9:963759. doi: 10.3389/fvets.2022.963759 (PMC9410625; doi:10.3389/fvets.2022.963759)
Supplement: Supplementary file 1 [file Data_Sheet_1.docx]

Supplementary Material

| **Treatment Group** | **Age** | **Weight (kg)** | **Total Ultrafiltrate Collected (μL)** | **Average Ultrafiltrate Volume Per Collection (μL)** |
| --- | --- | --- | --- | --- |
| Ultrafiltration probe | 12 | 513 | 5590 | 118.9 |
|  | 5 | 492 | 3970 | 97.32 |
|  | 4 | 405 | 2485 | 130.8 |
|  | 7 | 512 | 1520 | 104.0 |
|  | 4 | 405 | 4860 | 203.3 |
|  | 4 | 405 | 8040 | 187.0 |
| Control | 13 | 490 |  |  |

**Supplementary Table 1. Collected volume of tendon ultrafiltrate did not differ between horses.** Signalment of horses (n=7) undergoing creation of bilateral surgically induced SDFT injury is presented. Six horses underwent implantation of bilateral ultrafiltration probes with a single horse serving as control. No significant difference (p=0.944) was measured in total volume collected or average volume per collection of tendon ultrafiltrate between horses. Collection of fluid from the center of tendon lesions via percutaneous, ultrasound guided collection was unrewarding in the single control horse.


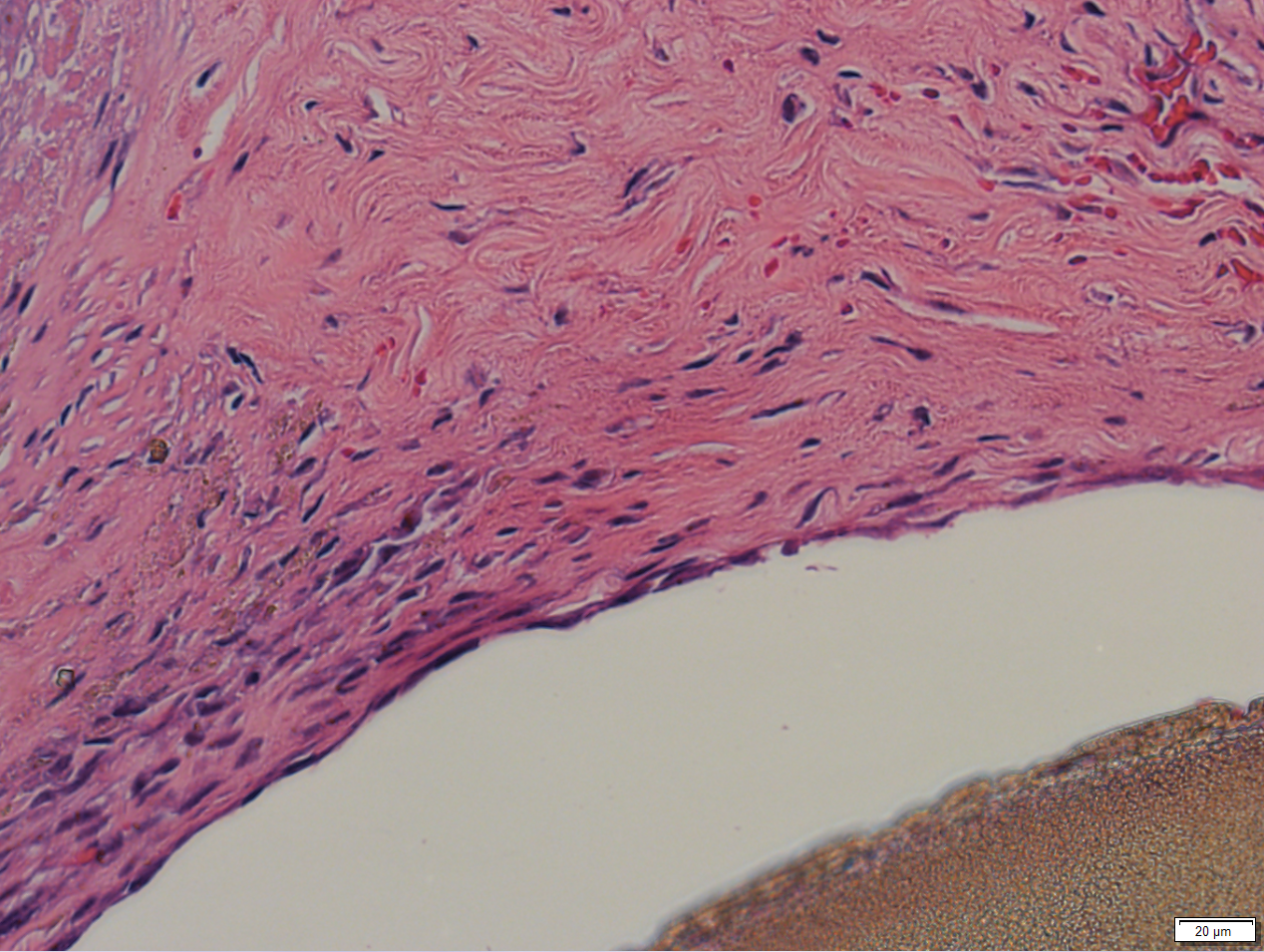

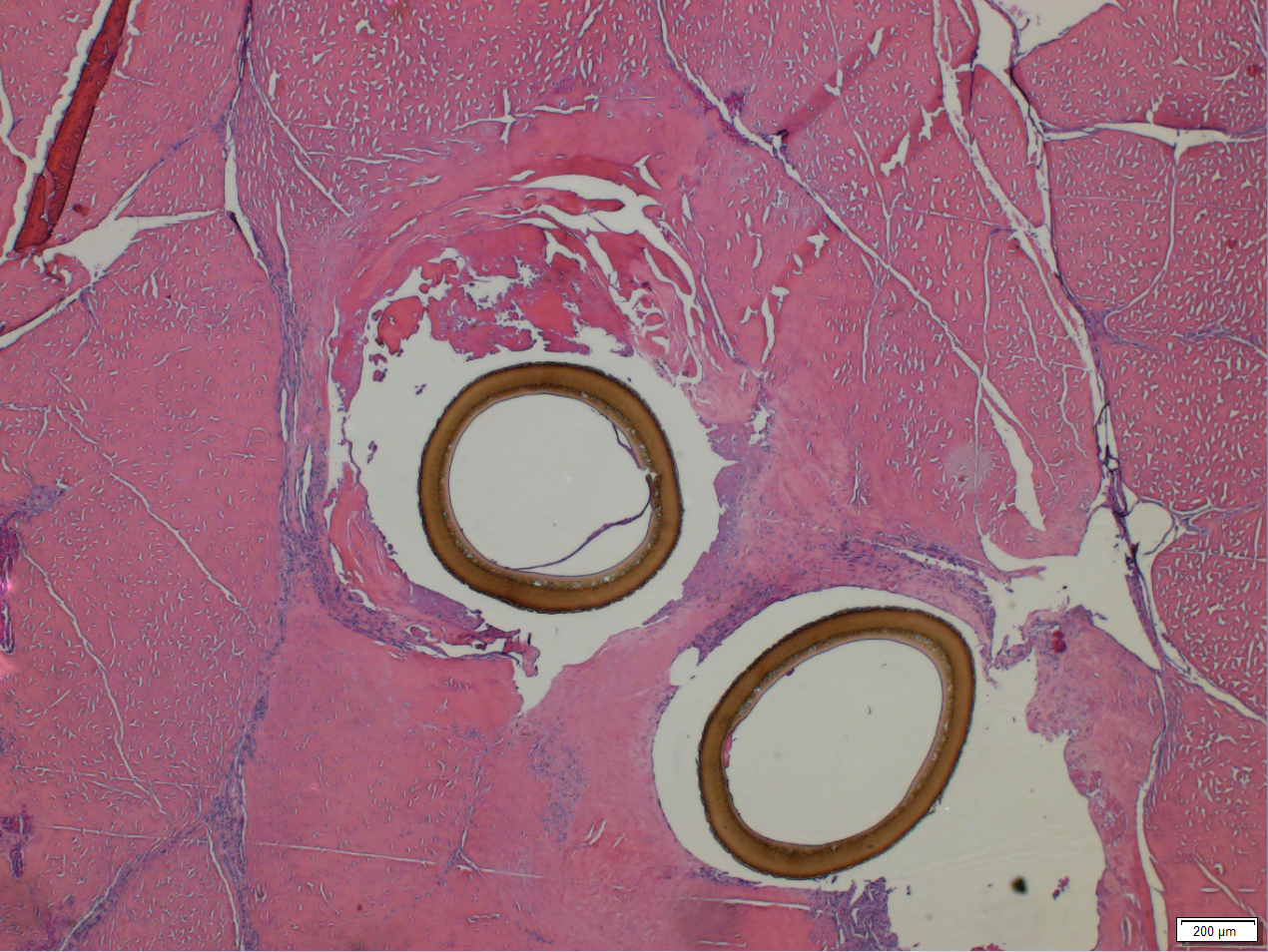


*

A

B

**Supplementary Figure 1. Ultrafiltration probe fibers did not induce histologic signs of foreign body reaction within the equine SDFT.** Histopathology with hematoxylin and eosin (H&E) staining at 4x (left) and 40x (right) confirmed the inert nature of the ultrafiltration probe fibers with presence of mildly reactive fibroblasts (arrow) surrounding the tan ultrafiltration probe fibers (asterisk) while lacking adjacent leukocyte infiltration.


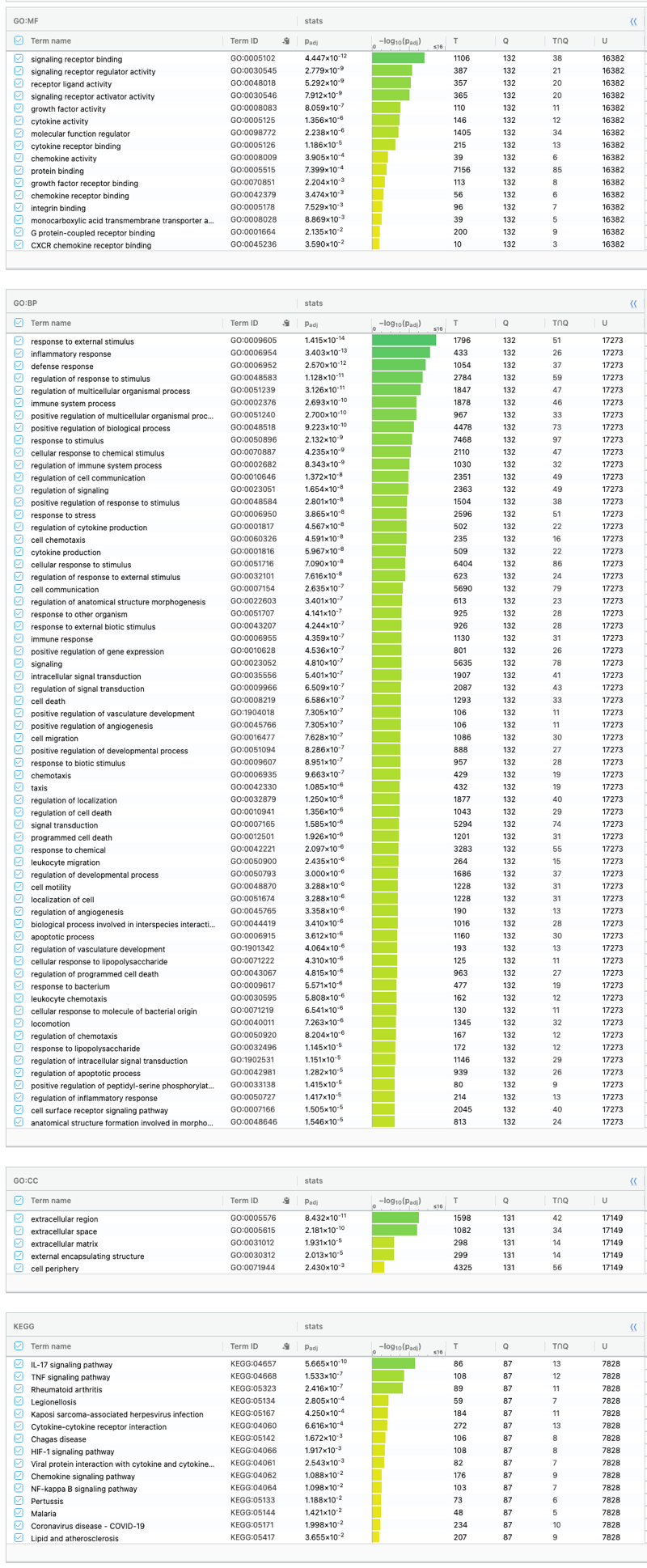
 **Supplementary Figure 2. GO term enrichment analysis for the top 100 upregulated genes in equine MSCs following IL-1β licensing.** Manhattan plots from g:Profiler illustrating GO term enrichment analysis for the top 100 upregulated DEGs in IL-1β licensed MSCs. MF: molecular function; BP: biological process; CC: cellular component)
